# Supplementary material for: Effect of Composition on the Physicochemical Properties of Cross-Linked Poly(sodium acrylate)/Sodium Silicate Hydrogels
Source: Materials (Basel). 2023 Nov 29;16(23):7422. doi: 10.3390/ma16237422 (PMC10707403; doi:10.3390/ma16237422)
Supplement: Supplementary file 1 [file materials-16-07422-s001.zip › materials-2708522-supplementary.pdf]

Appendix to the article entitled:

Effect of the composition on physicochemical properties of cross-linked poly(sodium acrylate)/sodium silicate hydrogels

Joanna Mastalska-Popławska[*1], Łukasz Wójcik[1], Piotr Izak[1], Damian Konaszewski[1]

[1]Faculty of Materials Science and Ceramics, Department of Ceramics and Refractories, AGH University of Science and Technology, 30-059 Krakow, Poland; lukwoj@agh.edu.pl (L.W.); izak@agh.edu.pl (P.I.); konaszew@student.agh.edu.pl (D.K.)

*Correspondence: jmast@agh.edu.pl (J.M.-P.)

1. Rheological measurements: gelation kinetics

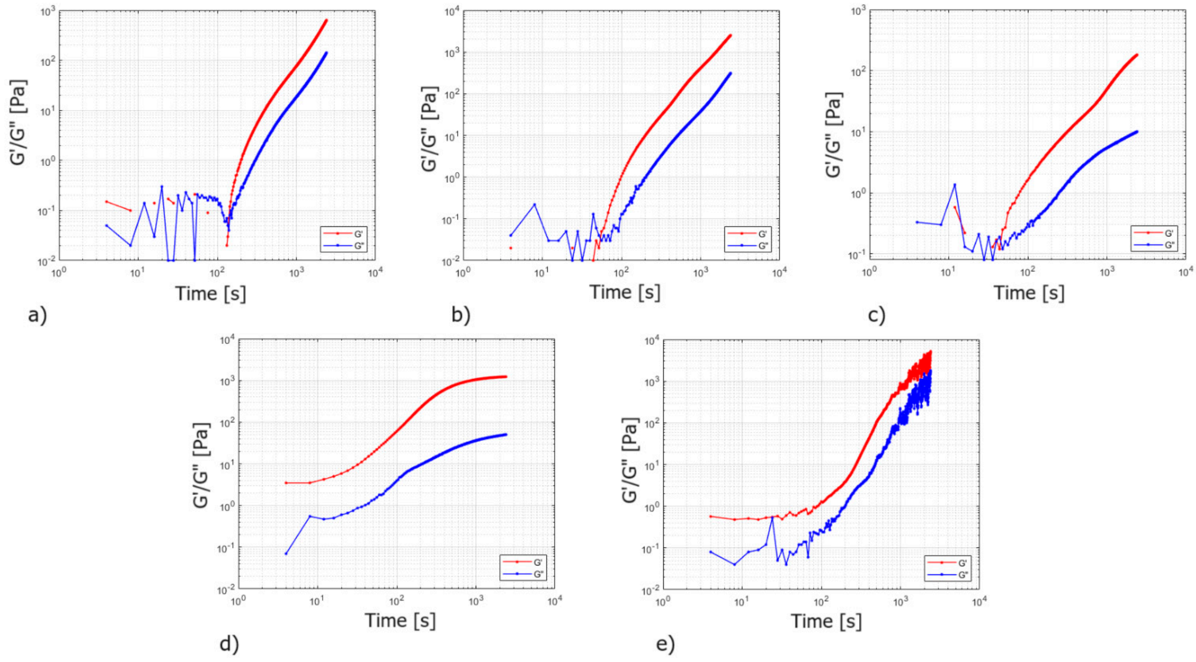

Figure S1. Graphs of gelation kinetics of silicate-polymer systems (20 wt.% of ANa aq.): a) 5%/1:9, b) 20%/1:9, c) 5%/1:1, d)20%/1:1, e) 5%/9:1.

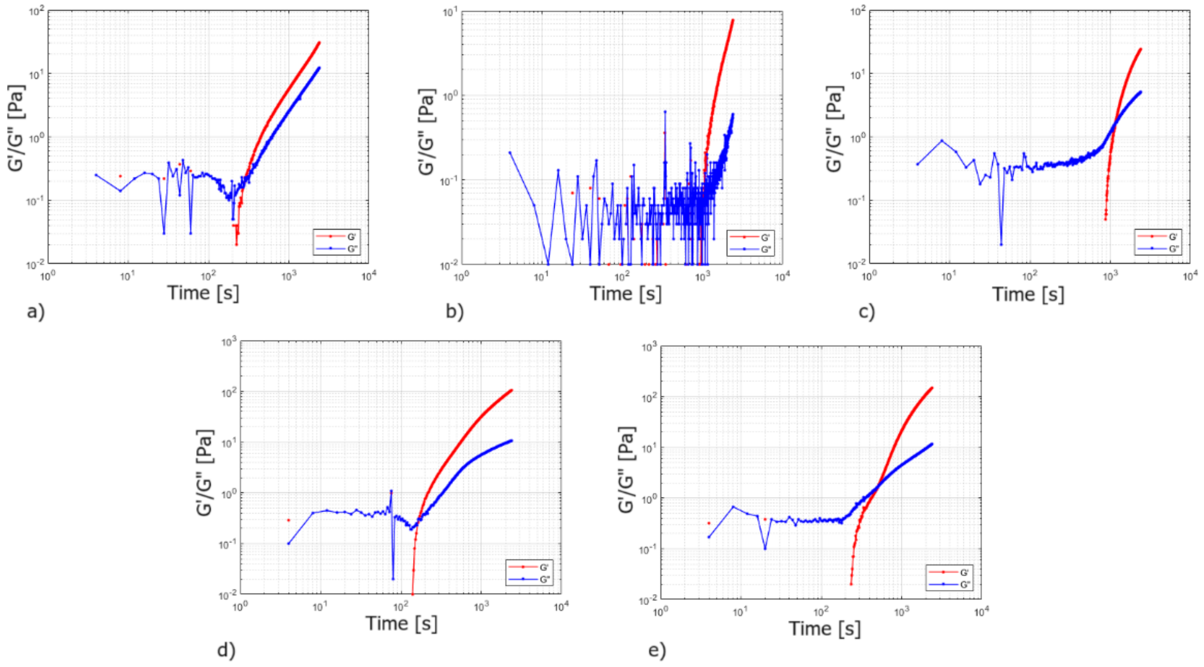

Figure S2. Graphs of gelation kinetics of silicate-polymer systems (15 wt.% of ANa aq.): a) 5%/1:9, b) 20%/1:9, c) 5%/1:1, d)20%/1:1, e) 5%/9:1.

2. Rheological measurements: oscillation measurements

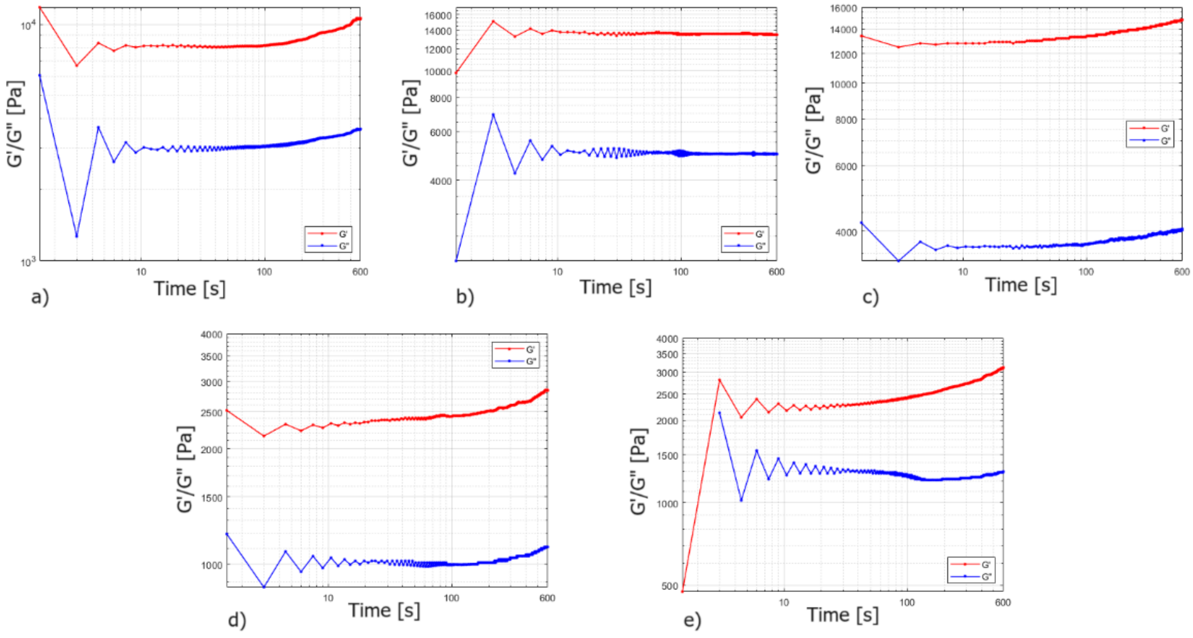

Figure S3. Graphs obtained during the oscillation tests (20 wt.% of ANa aq.): a) 5%/1:9, b) 20%/1:9, c) 5%/1:1, d)20%/1:1, e) 5%/9:1.

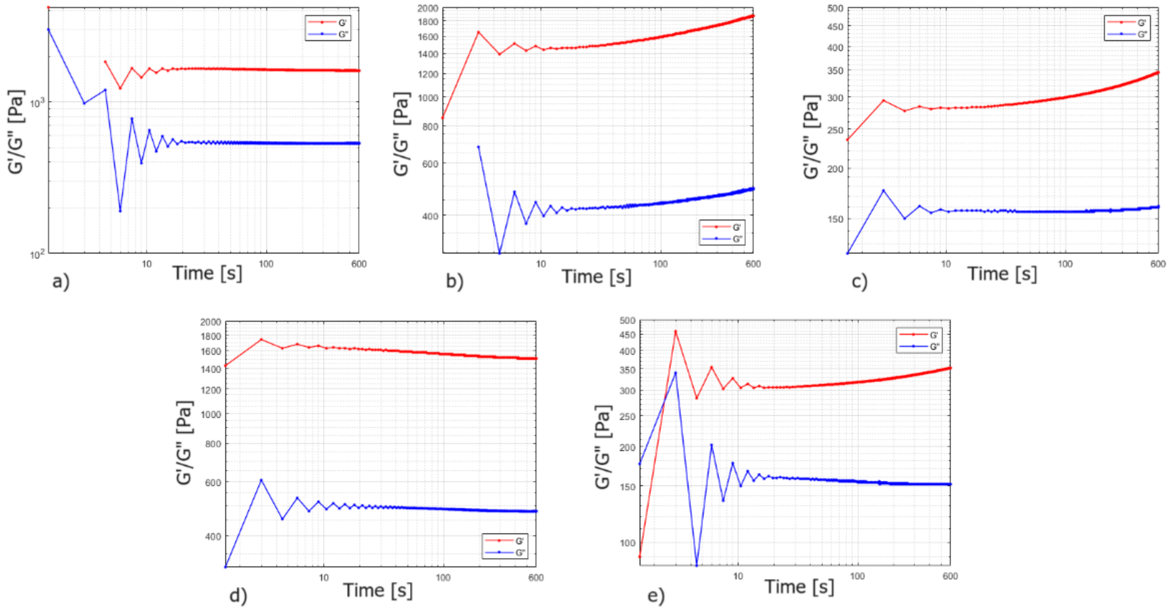

Figure S4. Graphs obtained during the oscillation tests (15 wt.% of ANa aq.): a) 5%/1:9, b) 20%/1:9, c) 5%/1:1, d)20%/1:1, e) 5%/9:1.

3. Fire tests

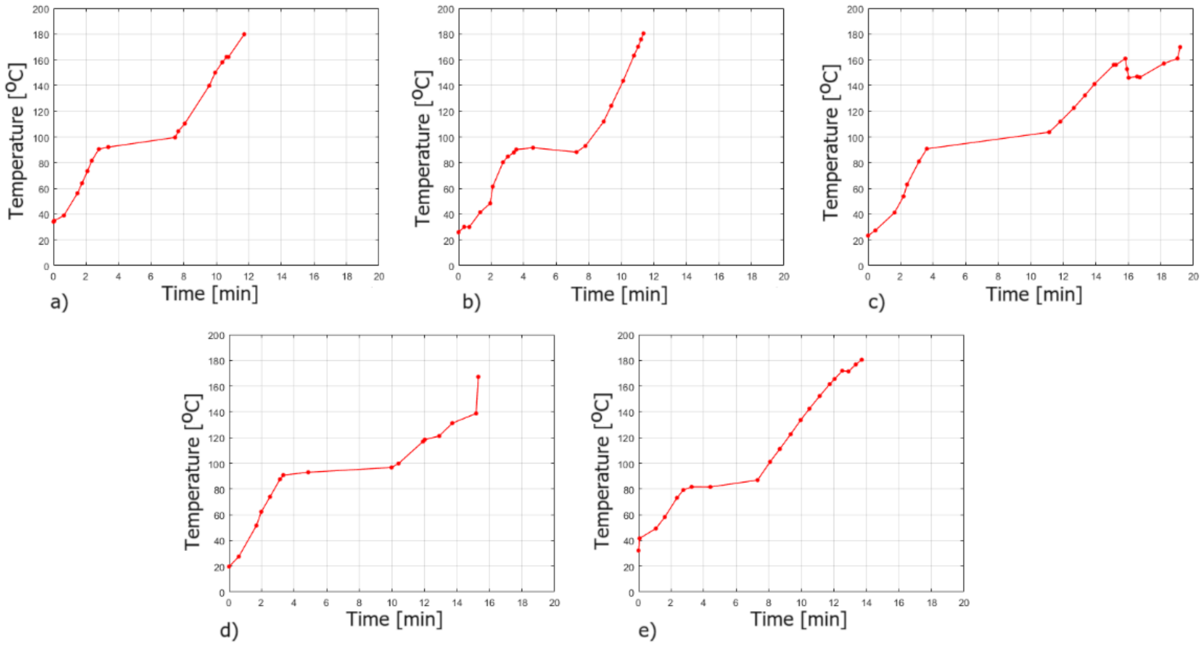

Figure S5. Temperature versus time during the fire test (20 wt.% of ANa aq.): a) 5%/1:9, b) 20%/1:9, c) 5%/1:1, d)20%/1:1, e) 5%/9:1.

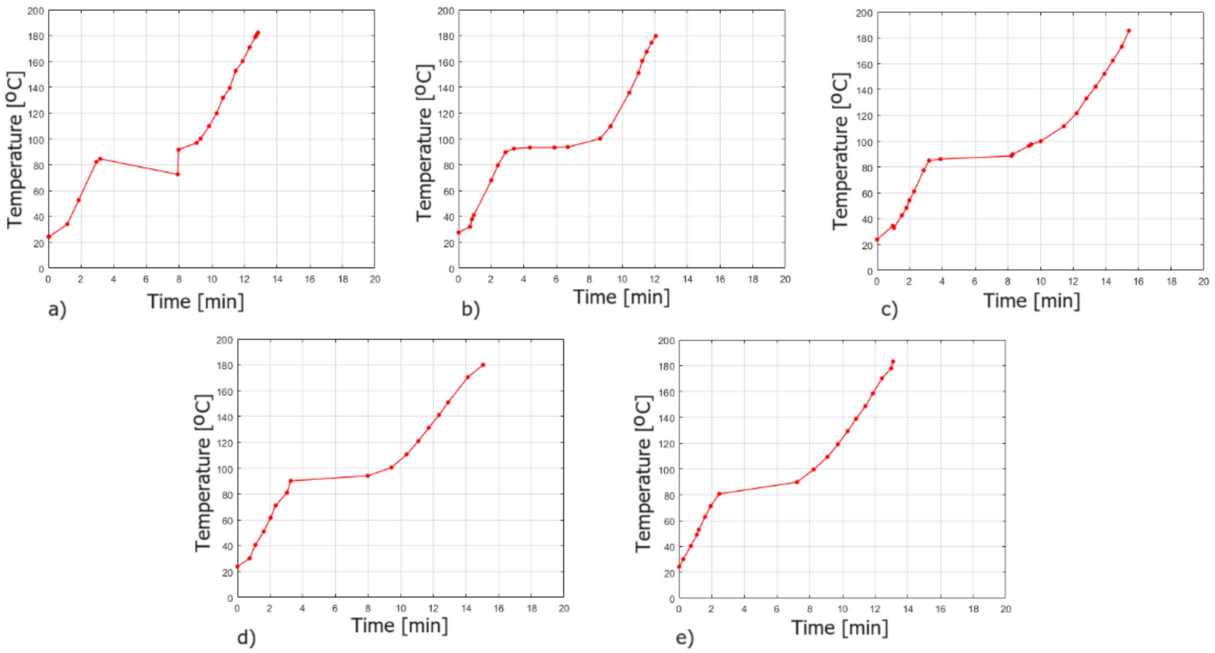

Figure S6. Temperature versus time during the fire test (15 wt.% of ANa aq.): a) 5%/1:9, b) 20%/1:9, c) 5%/1:1, d)20%/1:1, e) 5%/9:1.

4. Thermal analysis: TG/DSC analysis

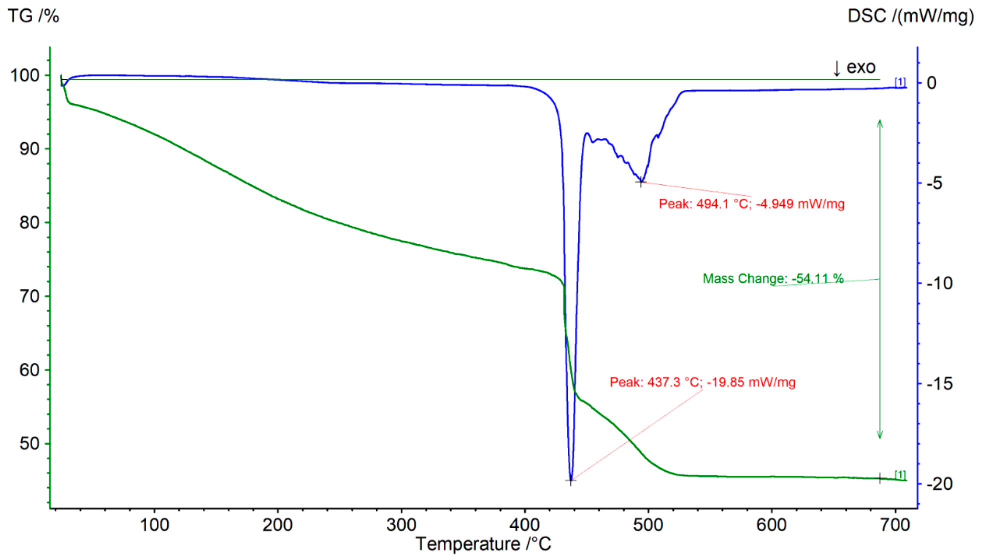

Figure S7. TG/DSC thermogram of sample 5%/1:9 (20 wt.% of ANa aq.).

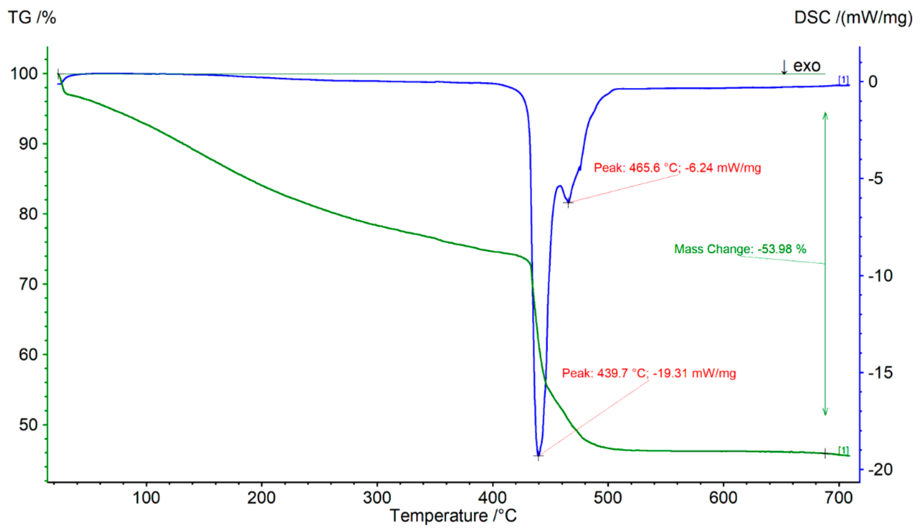

Figure S8. TG/DSC thermogram of sample 20%/1:9 (20 wt.% of ANa aq.).

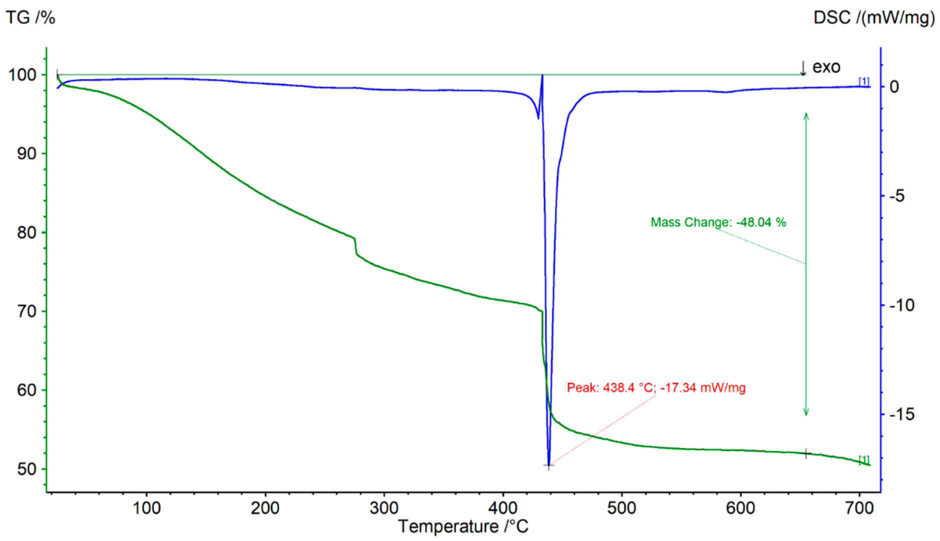

Figure S9. TG/DSC thermogram of sample 5%/1:1 (20 wt.% of ANa aq.).

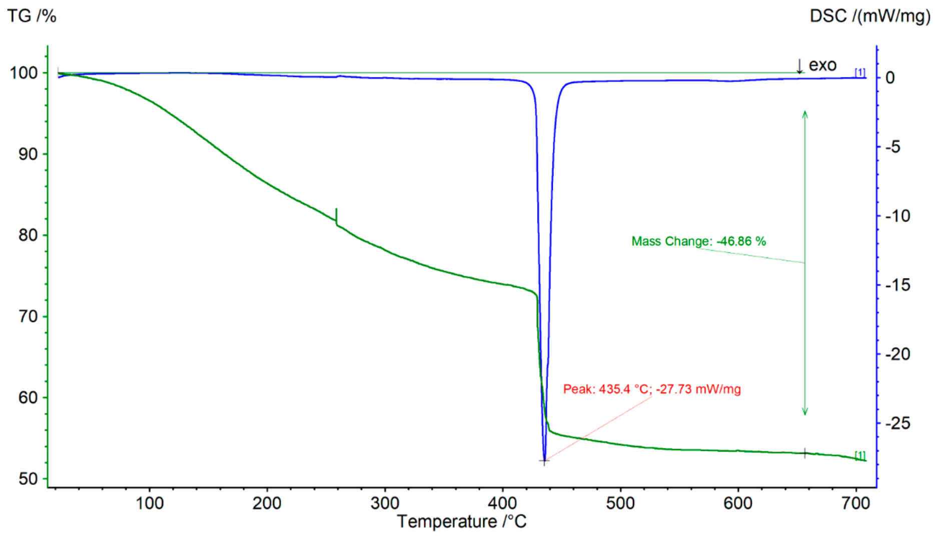

Figure S10. TG/DSC thermogram of sample 20%/1:1 (20 wt.% of ANa aq.).

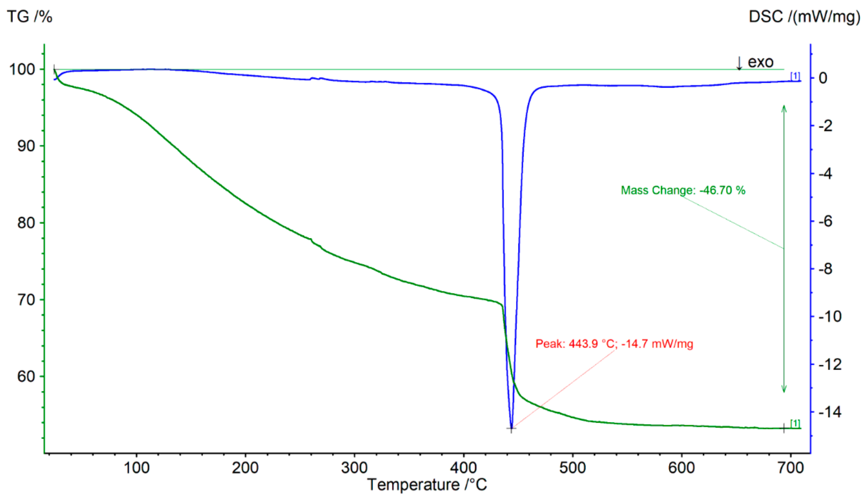

Figure S11. TG/DSC thermogram of sample 5%/9:1 (20 wt.% of ANa aq.).

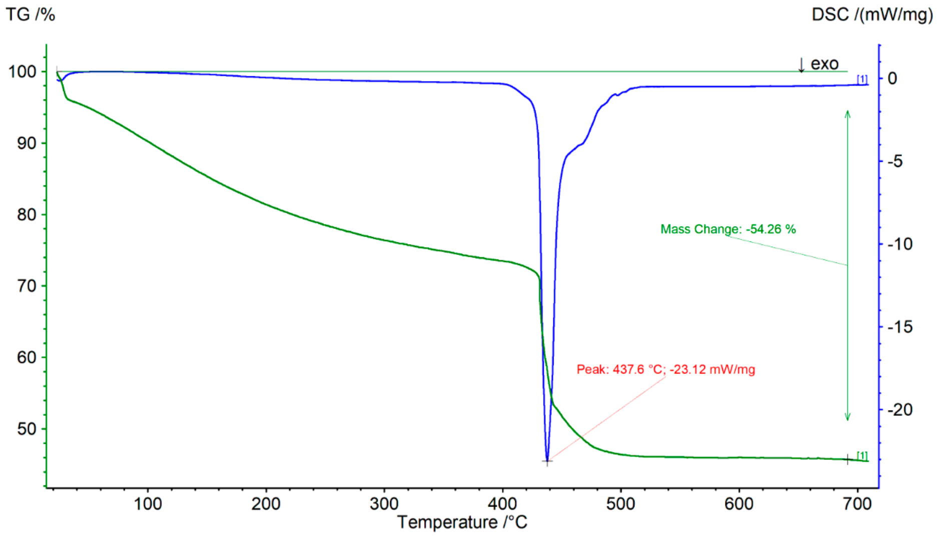

Figure S12. TG/DSC thermogram of sample 5%/1:9 (15 wt.% of ANa aq.).

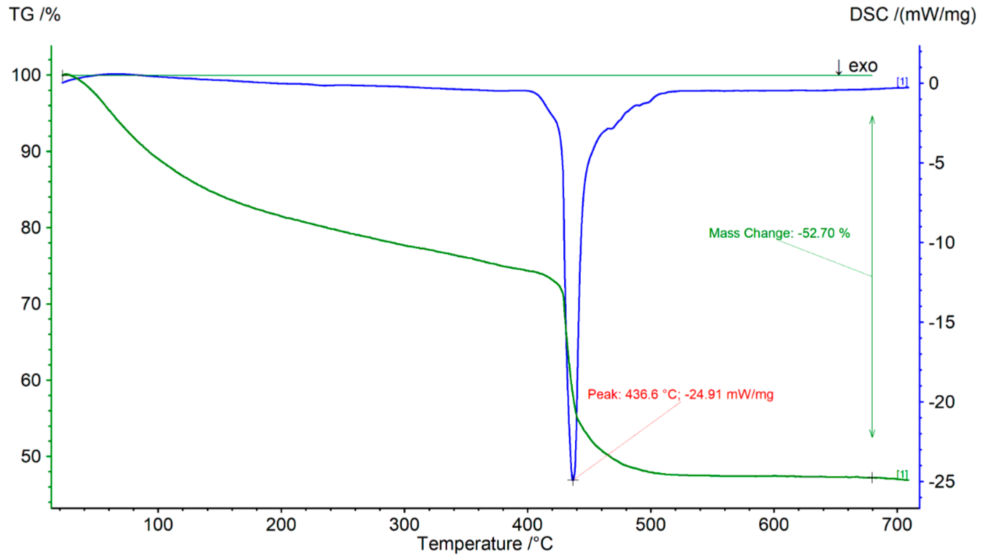

Figure S13. TG/DSC thermogram of sample 20%/1:9 (15 wt.% of ANa aq.).

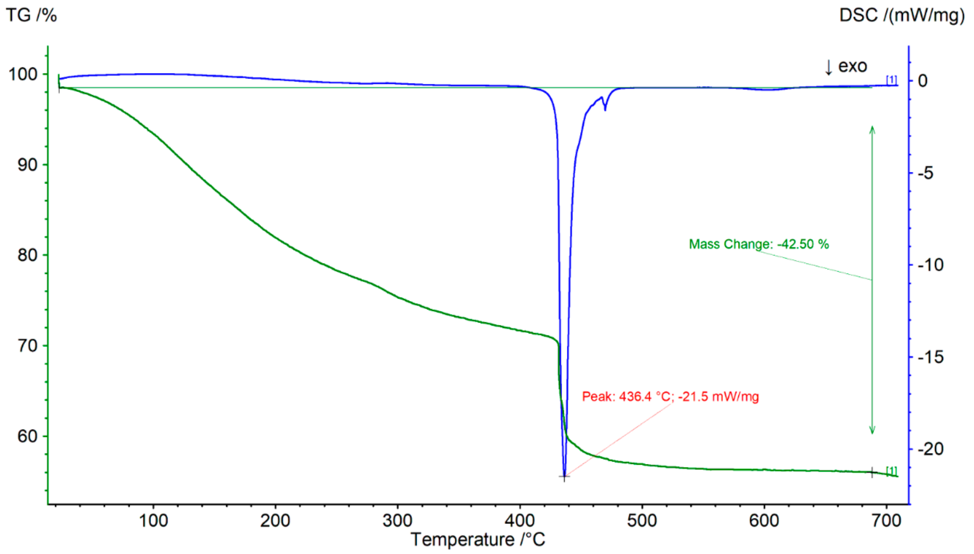

Figure S14. TG/DSC thermogram of sample 5%/1:1 (15 wt.% of ANa aq.).

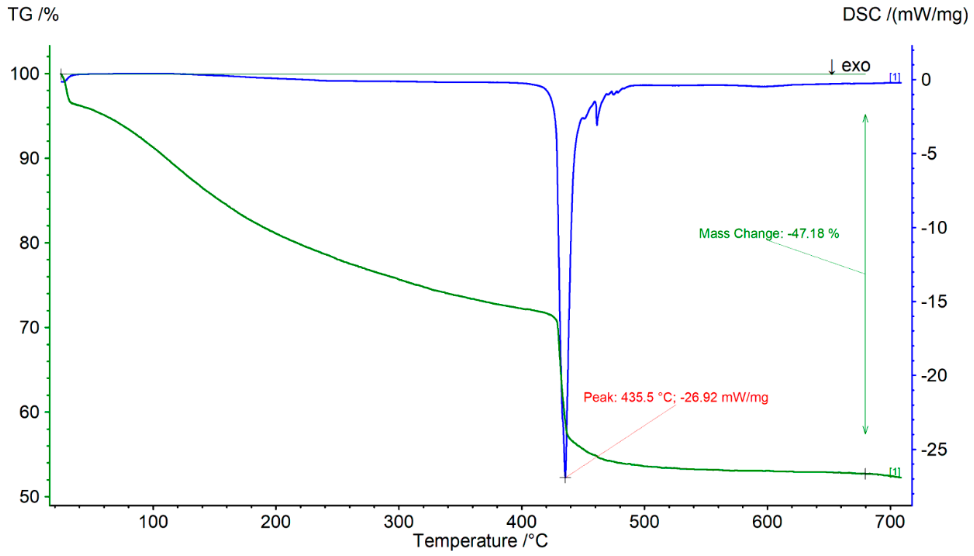

Figure S15. TG/DSC thermogram of sample 20%/1:1 (15 wt.% of ANa aq.).

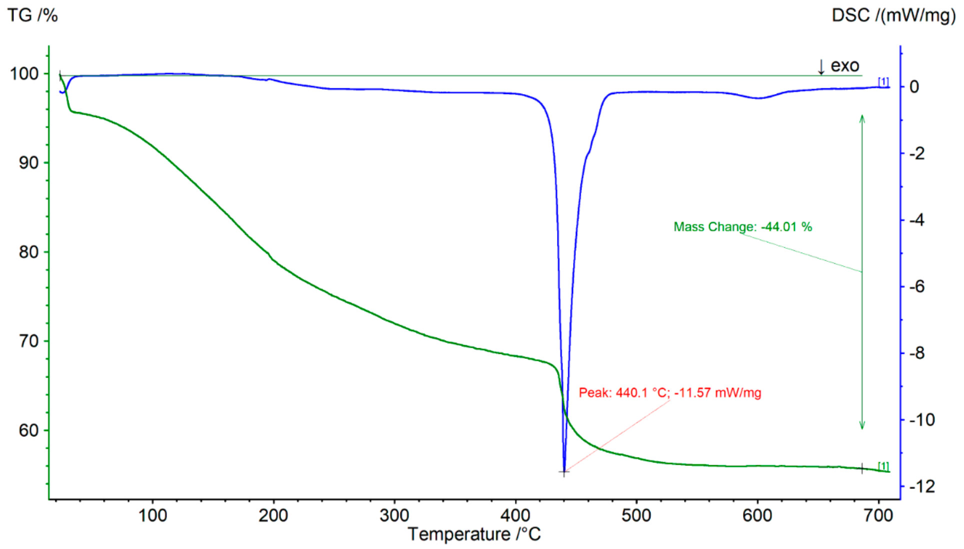

Figure S16. TG/DSC thermogram of sample 5%/9:1 (15 wt.% of ANa aq.).
